# Numerical Study on a Bio-Inspired Micropillar Array Electrode in a Microfluidic Device

**DOI:** 10.3390/bios12100878

**Published:** 2022-10-16

**Authors:** Chaozhan Chen, Bin Ran, Bo Liu, Xiaoxuan Liu, Jing Jin, Yonggang Zhu

**Affiliations:** 1 School of Science, Harbin Institute of Technology, Shenzhen, Shenzhen 518055, China; 2 School of Mechanical Engineering and Automation, Harbin Institute of Technology, Shenzhen, Shenzhen 518055, China

**Keywords:** micropillar array electrode, microchip-based electrochemical detection system, bionics design, numerical simulation, high sensitivity

## Abstract

The micropillar array electrode (µAE) has been widely applied in microchip-based electrochemical detection systems due to a large current response. However, it was found that amplifying the current through further adjusting geometrical parameters is generally hindered by the shielding effect. To solve this problem, a bio-inspired micropillar array electrode (bµAE) based on the microfluidic device has been proposed in this study. The inspiration is drawn from the structure of leatherback sea turtles’ mouths. By deforming a μAE to rearrange the micropillars on bilateral sides of the microchannel, the contact area between micropillars and analytes increases, and thus the current is substantially improved. A numerical simulation was then used to characterize the electrochemical performance of bµAEs. The effects of geometrical and hydrodynamic parameters on the current of bµAEs were investigated. Moreover, a prototypical microchip integrated with bµAE was fabricated for detailed electrochemical measurement. The chronoamperometry measurements were conducted to verify the theoretical performance of bµAEs, and the results suggest that the experimental data are in good agreement with those of the simulation model. This work presents a novel bµAE with great potential for highly sensitive electrochemical detection and provides a new perspective on the efficient configuration of the µAE.

## 1. Introduction

The microchip-based electrochemical detection system (µEDS) has drawn wide research attention in recent decades. Compared to conventional assay platforms, the µEDS offers many potentials, including small sample volumes, high automation and high sensitivity [1,2,3,4,5,6]. In the last decade, various of µEDSs were developed with respect to diagnostic applications such as disease detection [7,8,9] and real-time monitoring [10]. Nevertheless, a critical issue that arises from these applications of the µEDS is the use of a relatively low amount of the current response [11]. Undeniably, a good sensitivity is in question when the current response is weak. This severely restricts the broad application of the µEDS, by and large.

The working electrode (WE) is the most crucial element, since its features are directly related to the performance of the µEDS compared to the reference electrode (RE) and the counter electrode (CE). WEs in the µEDS often have two-dimensional (2D) planar forms of bands [12,13], disks [9,14], microarrays [6] and rings [15] and are directly integrated inside a microchannel. In addition to the planar configurations, the three-dimensional (3D) micropillar array electrode (μAE) has recently been reported as an emerging technology with a large current response [16,17,18,19]. In order to improve the electrochemical performance of the μAE, the effects of different geometric parameters (e.g., micropillar’s spacing, height and shape) on the current response of μAEs have been systematically studied [17,20,21,22,23]. However, these studies focused on improving the current response of the μAE by adjusting the geometric parameters. Furthermore, optimizing geometrical parameters is generally hindered by the shielding effect or the restricted construction region [24]. These issues have greatly impeded the sensitivity of the exiting μAE. In order to achieve µEDS with high sensitivity, there is a high demand for improving the electrochemical performance of the μAE.

**Figure 1:**
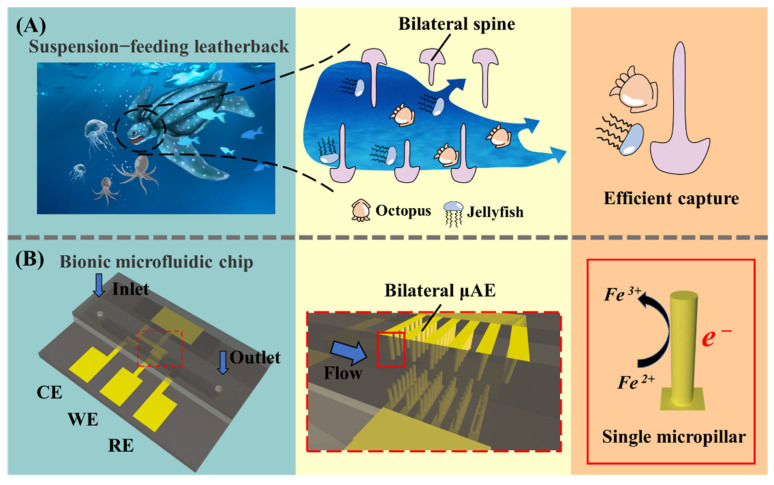
(**A**) The efficient capture mechanism of leatherback sea turtles, whose mouth and throat have bilateral projecting spines that can effectively grasp the free prey. (**B**) Electrochemical mechanism of the microfluidic chip. The micropillars are rearranged in bilateral sides of the microchannel, which increases the contact between the electrodes and analytes.

To further improve the electrochemical performance of the µEDS integrated with the μAE, a microfluidic chip integrated with a bio-inspired micropillar array electrode (bμAE) is proposed in this paper. The inspiration is obtained from leatherback sea turtles’ mouths. As the largest known turtle in the world, leatherback sea turtles swim in ocean waters to efficiently procure their food items, such as jellyfish and octopus [25]. Its mouth and throat have bilateral projecting spines that can effectively grasp the free prey (See Figure 1A). This suggests that a bilateral arrangement might be an effective way to improve the contact between spines and prey. Moreover, the functionality of this bilateral arrangement has rarely been considered before as one-sidedly arranged μAEs are usually easier to prepare. The one-sidedness often leads to a portion of the analyte failing to come into contact with the μAEs [18,26,27,28,29]. Considering this, it is critical to deform a μAE to rearrange the micropillars on bilateral sides of the microchannel, which increases the contact between the electrodes and analytes. 

Numerical simulations are a common and effective approach to understanding the performance of the µEDS integrated with bμAEs. By solving the Navier–Stokes equation, convention-diffusion equation and Butler–Volmer equation [30,31,32], conventional electrodes have been widely studied, such as microbands [33] and rings [15,34]. However, those research objects are usually simplified geometrical or mathematical models. When the working electrode is a complex μAE, the microchannel has a 3D flow, resulting in flow characteristics near the surface of electrodes, including edge effects [35,36] and tail effects [33,37]. These characteristics affect the electrochemical reaction processes at the surface of electrodes, and hence greatly influence the electrode’s current. Given this, this study adopted 3D simulations to investigate the electrochemical performance of the bµAE integrated into the microchannel.

In summary, this study proposed a bμAE based on the microfluidic platform and investigated the electrochemical performance of the microfluidic chip integrated with the bμAE. The effects of the micropillars of different spacings, heights, layouts, and shapes on the current of bµAEs were studied based on a numerical study. The tail effects in the microchannel were also analyzed using the dimensionless parameter of the current density ratio. Apart from optimizing geometric parameters, a microchip integrated with the bµAE was prepared using the standard microfabrication technology. Cyclic voltammetry (CV) and chronoamperometry (CA) electrochemical measurements were carried out to validate the numerical modeling method. This study presents a creative bµAE with a good potential for highly sensitive electrochemical detection and provides a new perspective on the configuration of the µAE.

## 2. Materials and Methods

### 2.1. Chemicals and Instrumentation

The hydrogen peroxide (H_2_O_2_), potassium ferricyanide (K_3_[Fe(CN)_6_]), potassium ferrocyanide (K_4_[Fe(CN)_6_]) and potassium chloride (KCl) were purchased from Macklin Biochemical Co. Ltd. (Shanghai, China). The negative photoresist SU-8 2075, SU-8 2025, and its developer solution were bought from MicroChem Corporation (Newton, MA, USA). The positive photoresist AZ5214 and its developer solution were purchased from Yancai Micro-nano Technology Co., Ltd. (Suzhou, China). Ultrapure water (18.2 MΩ·cm^−1^) and the phosphate buffer solution (PBS, 0.1 M, pH 7.4) were used for dilution. The sputtering deposition of the conductive metal layer was performed using the PD-400 (Pudivaccum, Wuhan, China). All electrochemical experiments (e.g., cyclic voltammetry and chronoamperometry) were carried out using the CHI 760E workstation (Shanghai Chen Hua Instrument Co. Ltd., Shanghai, China). The analyte was injected into the chip using the neMESYS 290N syringe (CETONI, Korbussen, Germany). The scanning electron microscopic (SEM) figures were caught using a ZEISS SUPRA 55 microscope (Carl Zeiss, Jena, Germany). The numerical model was established and solved using a precision 5820 workstation (Dell, TX, USA). All chemicals were analytical grade and used without further purification. All experiments were carried out at room temperature.

### 2.2. Configuration of Bionic Microchip

A bio-inspired micropillar array electrode (See Figure 1B) based on the microfluidic chip was developed to achieve highly sensitive electrochemical detection. The microchip contained a bμAE acting as a WE, a CE, and a RE. The RE and CE were put on the two sides of the WE, and Ag/AgCl ink was coated on the tip of the RE. These three electrodes were inserted into a microchannel, with a height of 500 µm. The bμAE-containing micropillars was located in a specified projected region (1.5 × 2.5 mm^2^). The micropillars with different heights (50, 100, 150, 200 and 250 µm), spacing (150, 200 and 250 µm), layouts and shapes were selected for simulation.

### 2.3. Simulation Method of BμAE

For the bμAE based on the microfluidic chip, we assumed this to be a one-electron, fully reversible electrochemical system in this study. At the surface of the working electrodes, the electrochemical reaction is listed below (Equation (1)):

(1) $$A+ne^{-}⇄B$$

where *A* and *B* are the oxidized and reduced substances, respectively. *n* is the number of electrons involved, and there is an electrochemical reaction with a single-electron transfer. 

The mass transport of the oxidized and reduced substances to the surface of electrodes in the solution includes the convection term and the diffusion term (Equation (2)).

(2) $$\frac{\partial C}{\partial t}=D\nabla^{2}C-\vec{u}\cdot \nabla C$$

where *t* is the time in seconds, C is the concentration of the analyte in mol·cm^−3^, D is the diffusion coefficients of oxidized and reduced substances in cm^2^·s^−1^ and u is the flow velocity. 

At the surface of electrode, the chronoamperometric current is calculated by Butler-Volmer equation (Equation (3)) [21]:

(3) $$i=nFS(k_{f}C_{O}(t)-k_{b}C_{R}(t))$$

where *F* is the Faraday constant, *S* is the area of the electrode, *C_O_(t)* and *C_R_(t)* are the concentration of the redox substances at time *t*, respectively. *k_f_* and *k_b_* are the heterogeneous electron transfer rate constant given by Equations (4) and (5) [21].

(4) $$k_{f}=k_{0}\exp\left(-\alpha \frac{F}{RT}(E-E^{0^{\prime }})\right)$$

(5) $$k_{b}=k_{0}\exp\left(\left(1-\alpha )\frac{F}{RT}(E-E^{0^{\prime }}\right)\right)$$

where *T* is the temperature, *α* is the charge transfer coefficient, *k_0_* is the standard transfer rate constant, *E^0′^* is the formal potential of the redox substances, *R* is the gas constant and *E* is the potential applied to the electrode.

As the microchannel integrated with the bμAE is symmetric, domains corresponding to half the fluid region were built to simulate the response of bμAEs with various dimensions. The computational domains were discretized using the hexahedral mesh. Figure S1 displays the diagrams of the domain and grid. The steady-state current response of planar electrodes, μAEs, and bμAEs with varying configurations was investigated. The geometric parameters of these electrodes are shown in Table 1. Based on the parameters of μAEs in existing studies [16,17,18,38], the center-to-center spacing between two adjacent micropillars and the height of the micropillars varied from 150 to 250 μm and 50 to 250 μm, respectively. The radius of the micropillar was set as 50 μm. Micropillars array was distributed in a designed projected area (1.5 × 2.5 mm^2^). Furthermore, in order to obtain the steady-state current, flux-free conditions were used at all boundaries except the electrode surface. The boundary of the inlet and outlet were the constant analyte concertation of 5 mM and the atmospheric pressure, respectively. The boundary conditions and control equations are exhibited in Table 2. The simulation was carried out using the Chemical Reaction Engineering module in the finite-element software COMSOL Multiphysics 5.4.

### 2.4. Fabrication of Bionic Microchip

**Figure 2:**
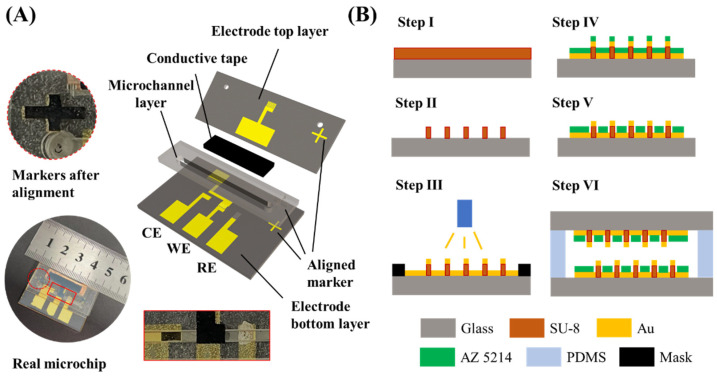
(**A**) The explosion view of the bionic microfluidic chip and the figure of the real chip. (**B**) The fabrication process of the bionic microfluidic chip.

Figure 2A shows an explosion view of the developed bionic microchip. This chip has an electrode bottom layer with a RE, a CE and half of bμAE, an electrode top layer with another half of bμAE, a conductive tape layer, and a microchannel layer with a 25 mm long main channel (width of 1.5 mm and height of 500 μm). The microchannel layer was prepared by laser cutting the double-sided tape to form a microchannel. The microchannel layer was used to bond the electrode top and bottom layers. The conductive tape with a length of 25 mm and a width of 6 mm allows for the bottom layer and the top layer of the bμAE to be electrically connected. 

The bμAEs were fabricated in a cleanroom using the standard micromachining technology described in published papers [21,39,40,41,42]. The fabrication process is summarized in Figure 2B. A glass substrate was placed as electrode support, which was previously cleaned with deionized water to eliminate particles. Next, the SU-8 photoresist layer was spin-coated on the glass substrate (step Ⅰ). Afterward, the glass coated with the SU-8 film was exposed to ultraviolet light under a mask and then heated at 65 °C for 3 min. After entirely developing the SU-8 photoresist (step Ⅱ), the SU-8 pillars were then covered using a steel mask and placed in a sputter to deposit a conductive membrane on the SU-8 pillars. Firstly, the chromium film was deposited on SU-8 pillars as the adhesion layer with a thickness of near 50 nm, and then the gold film was subsequently deposited with a thickness of near 250 nm to form the conducting membrane (step Ⅲ). Thereafter, a 4 μm positive photoresist (AZ 5214) layer was spin-coated on the gold layer prepared in step Ⅲ. The positive photoresist was exposed to UV light on a mask aligner and then was developed completely to acquire half of bμAE (step Ⅴ). The unexposed positive photoresist prevents the gold layer from contacting the solution, thus determining the total surface area of the bμAE. Finally, the developed bottom layer, microchannel layer, and top layer were sequentially combined by matching the reserved aligned markers (See the inset of Figure 2A). The above alignments were performed with a microscope, and the combined chip is shown in Figure S2A. Furthermore, the dye solution was used to illustrate the feasibility of the microchip based on the adhesion of the double-sided tape. It can be seen in Figure S2B that there is no leakage when the dye solution is injected into the chip at the flow rate of 100 µL·min^−1^.

### 2.5. Experiments of Electrochemical Detection

The verification of the numerical model was performed in the solution containing 5 mM K_3_[Fe(CN)_6_]/K_4_[Fe(CN)_6_] and 0.1 M KCl. Figure S2C shows the schematic diagram of the detection system. The potential of +0.4 V was used as the working electrode’s potential for CA experiments. The CA test was performed at a potential of +400 mV for 60 s. The current was recorded at 50 s when the current was steady. The analyte solution was injected into the microfluidic chip with a flow rate range from 0 to 30 µL·min^−1^. Electrochemical measurements were repeated at least three times.

## 3. Results and Discussion

### 3.1. Effect of Flow Rate and Spacing

The μAE and bμAE were created to produce a stronger electrical signal because they have a much larger surface area than planar electrodes with the same projected area. The surface area of the bμAE was strongly influenced by their dimensions, such as spacing, diameter, and height. The effects of the spacing (*d*) between two adjacent micropillars on the electrochemical performance of the bµAE were investigated, with both the planar electrode and the conventional μAE being taken into consideration as a reference. In this chapter, the radius and height of the micropillars were all fixed to 50 µm and 150 µm, respectively.

**Figure 3:**
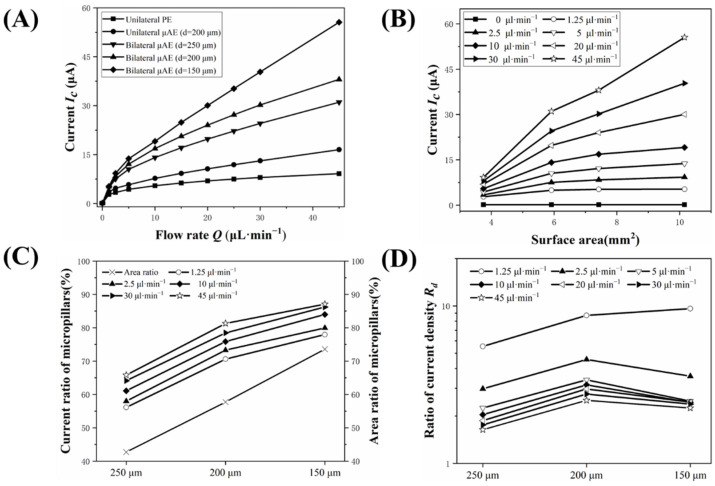
(**A**) Current responses of unilateral PE, unilateral μAE and bilateral μAEs with various spacings at different flow rates. (**B**) Current responses of PE, bilateral μAEs with various surface area at different flow rates. (**C**) Current and area ratios of bilateral μAEs with various spacings at different flow rates. (**D**) Ratios of the current density between the first and last row of micropillars.

The numerical simulation was employed to study the effects of spacing on the electrochemical performance of bµAEs. Figure 3A shows the current of the PE, the μAE, and bμAEs with different spacings at various flow rates. This indicates that a higher flow rate often causes a larger current regardless of electrode type, especially for bμAE. Moreover, the bμAE achieves a larger current response than µAE for the same total surface area, illustrating the advantages of the bilateral structure arrangement. For example, when the flow rate of 10 µL·min^−1^ is applied, the current of bµAE (16.8 μA) with the spacing of 200 µm is 2.18 times that of the µAE with the same spacing (7.7 μA). Furthermore, the current responses of the PE and bμAEs with different surface areas (caused by the varying spacing) are shown in Figure 3B. Expect for the stationary condition, the bμAE always yields a larger current than PEs. For example, when the flow rate varies from 1.25 to 45 µL·min^−^^1^, the current of bµAE with the 200 µm spacing is 1.9–3.4 times that of PEs. As a supplement, the concentration distribution in the microchannel at different flow rates is given in Figure S3. Figure S3 shows that the blue area of low concentration shrinks with the flow rate increases. Higher flow rates eventually result in a larger current because they not only raise the concentration gradient of analytes near the surface of bμAE but also provide more analytes for the electrochemical reaction. However, for practical applications that require only low flow rates (<10 µL·min^−1^), small spacings do not further improve current response due to the almost complete reaction of electrodes and reactants. Therefore, we should choose the appropriate spacing according to the applied flow rate.

The influence of micropillars on the total current of the bμAE was numerically studied. The planar base area and the area of micropillars constitute the surface of bμAE. These two components work together to provide the overall response current. Figure S4 shows the current of the three components (i.e., the micropillar, the planar base and total surface area) of μAE and bμAE with a spacing of 200 µm at various flow rates. When the flow rate ranges from 1.25 to 45 µL·min^−1^, the current of these three components of the bμAE is always larger than that of the μAE. For instance, the current response of micropillars of the bμAE is 1.4 and 2.3 times that of micropillars of the μAE at 1.25 and 45 µL·min^−1^, respectively. This improvement demonstrates that the bilateral arrangement of μAE can effectively enhances the current responses of both micropillars and planar bases, resulting in a total current response improvement. Moreover, to study the micropillars’ contribution to the total current, we define the surface area ratio as the surface area of micropillars divided by the total surface area of bμAEs. The current ratio is also defined as the current of micropillars divided by the total current of bμAEs. Figure 3C shows the current ratio and the area ratio of bµAE with different spacings of micropillars. The result reveals that the current ratio increases with the flow rate and most of the current derive from the micropillars’ surface. For example, the micropillar current of the bμAE with a spacing of 200 µm provides approximately 70% of the total current of bμAE when the flow rate is 2.5 µL·min^−1^. Besides, the current ratios of the above bμAEs are higher than the area ratio in the range of flow rates from 0 to 45 µL·min^−1^, illustrating the significant role of micropillars in yielding a large current.

The tail effect is that upstream micropillars would influence the electrochemical performance of downstream micropillars of the bμAE due to the consumption of the analyst in the microchannel [17,33]. In order to study the tail effect, the current of the first and last row of micropillars was recorded and plotted in Figure S5. The tail effect exists both in cases of the bμAE and μAE, with a spacing of 200 μm. However, when the flow rate ranges from 2.5 to 45 µL·min^−1^, the current of both the first and last row of micropillars is always larger than that of μAEs. This indicates that the bilateral arrangement of bμAE allows for the analyte and electrode to react more fully, resulting in a large current. In addition, to study the tail effect of the bμAE with different spacings, we defined the current density ratio (R_d_) by the proportion of the current density of the first row of micropillars to that of the last row of micropillars. The variation trend of the current density ratios of bμAEs is displayed in Figure 3D. As the flow rate and the spacing decrease, the tail effect usually becomes more pronounced. Furthermore, the concentration downstream of analytes becomes lower with the consumption upstream (See Figure S6), resulting in a decrease in current of the last row of the micropillars. The tail effect limits the advantages of the increased surface area’s positive effects on the bμAEs. In addition, reducing the spacing exacerbates the tail effect. It is safe to conclude that the large spacing cannot take advantage of the large surface area of bμAEs, while small spacings fail to improve the current further due to the tail effect.

### 3.2. Effect of Micropillar Height

The height (*h*) of the micropillar is another critical factor affecting the surface area of the bμAE. According to our previous studies [16,17], increasing the height of micropillars directly increases the surface of the electrode and leads to a large current response. Predictably, the electrochemical performance of bμAEs is strongly correlated with the height of micropillars. Thus, we focus on the influence of the pillar height on the current response of bμAEs in this chapter.

**Figure 4:**
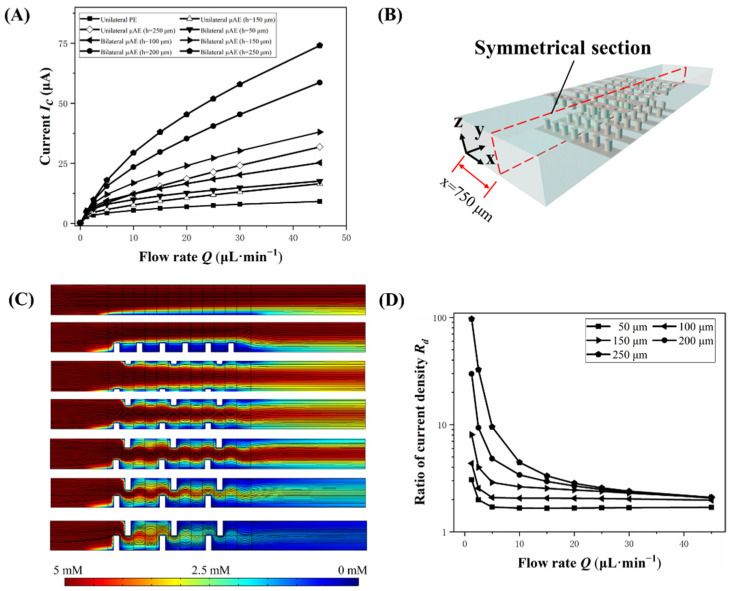
(**A**) Current responses of PEs, unilateral μAEs and bilateral μAEs with micropillars of different heights under varying flow rates. (**B**) The schematic diagram of the symmetrical section. (**C**)The concentration distribution of PEs, μAEs and bμAEs at the flow rate of 10 μL·min^−1^ (from top to bottom): The planar electrode; The unilateral μAE with 150−μm micropillars; The bμAE with 50−μm micropillars; The bμAE with 100−μm micropillars; The bμAE with 150−μm micropillars; The bμAE with 200−μm micropillars; The bμAE with 250−μm micropillars. (**D**) Ratios of the current density between the first and last row of micropillars.

As shown in Figure 4A, the electrochemical performance of the bμAE with different micropillar heights is investigated. The spacing of the micropillars is all set as 200 µm. It can be seen that higher micropillar heights lead to large current responses, no matter the μAE or the bμAE. However, at the same micropillar height, the current of bμAEs is always larger than that of the μAE. For instance, the current of bµAE (29.4 μA) with a micropillar height of 250 µm is 2.40 times that of the µAE (12.3 μA) with the same micropillar height when the rate of analyte injection is 10 µL·min^−1^. This improvement clearly illustrates the advantages of the bilateral arrangement of bµAE. Moreover, even if the height of micropillars is low, there is still a big difference between the current response of bμAEs and that of μAEs. Actually, when the micropillars are too low to immerse in the diffusion layer, no significant difference in the current between μAEs and the planar electrode could be observed [17]. However, our results reveal that the bilateral arrangement allows for the low micropillar to weaken the effects of the diffusion layer near the surface of electrodes. To study the difference in diffusion layers around the electrode’s surface between μAEs and bμAEs, Figure 4B–I shows the concentration distributions of different electrodes on the symmetrical section at 10 µL·min^−1^. Unlike the micropillars of μAEs immersed in the diffusion layer, the diffusion layer of the bμAE is thinner and usually shows a distinct profile. This may be because the bilateral arrangement of bµAE indirectly increases the spacings between adjacent rows of micropillars array, resulting in less influence of the diffusion overlay from adjacent rows of micropillars. In addition, as the height of micropillars increases from 50 to 250 μm (half the height of the microchannel), most of the analytes were consumed by the bμAE, indicating the importance of the height of micropillars. However, when the height of the micropillars is higher than half of the microchannel, the assembly of bμAEs would become an issue. Therefore, on the premise of realizing the assembly of bμAEs, we should select high micropillars in order to pursue good electrochemical performance of bμAEs.

On the other hand, the tail effects in bµAEs with different heights of micropillars are studied. As demonstrated in Figure 4D, as the flow rate increases from 0 to 40 μL·min^−1^, the tail effect is reduced, and the bµAE with the relatively high micropillars (>100 μm) displays almost the same current density ratio. Besides, when the applied flow rate is low, high micropillars lead to a large current density ratio due to most of the analytes being consumed by first few rows of micropillars and the few residual analytes are subsequently contacted with the last row of micropillars. In order to explain the significant tail effects when the flow rate is relatively low, the concentration distribution of the bμAEs with high micropillars is shown in Figure S7A–E. Figure S7 clearly shows that as the flow rate decreases, the last row of micropillars is exposed to a lower concentration of analytes. It is thus deduced that high micropillars aggravate the tail effect to a great extent at a low flow rate.

### 3.3. Effect of Micropillar Layout

**Figure 5:**
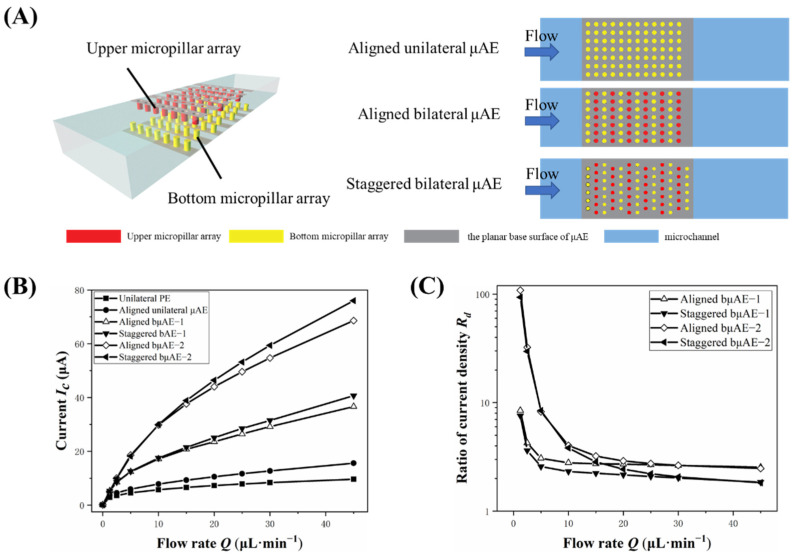
(**A**) The schematic diagram of the layout of the unilateral μAE and the bμAE. (**B**) Current responses of the PE, the unilateral μAE and bilateral μAEs in different layouts under varying flow rates; (**C**) The current density ratios of these bμAEs in different layouts under varying flow rates.

In addition to the change in surface area (caused by varying spacing and micropillar height), the spatial distribution of micropillars also affects the contact of the analyte with electrodes. When micropillars are arranged differently on the same surface area, the microchannel has a complex flow, resulting in a large variation in the current response [17]. Thus, the different layouts should be considered to optimize the response current of bµAE. In this work, two kinds of bµAE layouts were designed to determine the effect of the arrangement of micropillars in a horizontal direction. On the horizontal plane, the same number of micropillars are arranged in either staggered or aligned layouts, as shown in Figure 5A. In order for all electrodes to have the same projected area, the parameters of the PE, µAEs and bµAEs used for this section are listed in Table S1. 

The current of the PEs, μAEs and bμAEs with different layouts of micropillars are shown in Figure 5B. In the cases with the flow rate ranging from 1.25 to 10 µL·min^−1^, there is no obvious difference between the currents of bμAEs with different layouts of micropillars regardless of the pillar heights. However, when the flow rate ranged from 15 to 45 µL·min^−1^, the current of bµAEs with staggered layouts was higher than those with aligned layouts. As shown in Section 3.1, we quantitively discussed the current and the concentration distribution of the bµAE with a staggered layout. For the bµAE with the aligned layout, the analytes (blue area) that underwent the upstream reaction continued to participate in the subsequent reaction on the same profile plane (see Figure S8A–D). Compared to an aligned layout, micropillars in a staggered layout could be weakly influenced by adjacent rows of micropillars due to the mass transfer on different profile planes. To investigate the influence of different layouts on the current of the downstream rows of micropillars, we recorded the current of the second row of micropillars for analysis. Figure S8E displays the current of the second row of micropillars in both staggered and aligned layouts. From the figure, when the flow rate ranges from 1.25 to 45 µL·min^−1^, the current of the second row of micropillars in a staggered layout is always larger than that of the micropillars in an aligned layout, illustrating the disadvantage of mass transfer on the same profile plane. A staggered layout allows for the mass transfer of the analyte to occur in different profile planes between adjacent micropillars rows, thus improving the current. Therefore, it could be concluded that we should choose the staggered layout to yield a large current. 

The tail effect in the bµAE with different layouts was then investigated in Figure 5C. There is a similar current density–flow rate curve when the flow rate is low. When the flow rate is in the range from 10 to 45 µL·min^−1^, the current density ratios of bµAEs with aligned layouts are always larger than that of bµAEs with staggered layouts. This difference might be because micropillars in staggered layout could be less affected by the adjacent rows of micropillars, resulting in an increasing current of the last row of micropillars. Figure S8F shows the current of the micropillars’ first and last rows with different layouts. It seems that the current of the first row of micropillars with aligned layouts is almost the same as that with staggered layouts, regardless of the flow rate. As a comparison, when the flow rate increases to 10 µL·min^−1^, the current of the last row of micropillars with staggered layout is clearly larger than that with aligned layouts, demonstrating the enhanced mass transfer near the last row of micropillars with staggered layouts. Therefore, it could be inferred that the effect of the bµAE with staggered layouts improve the electrochemical performance by reducing the tail effect, differing from that of increasing the surface area of the bµAE.

### 3.4. Effect of Micropillar Shape

**Figure 6:**
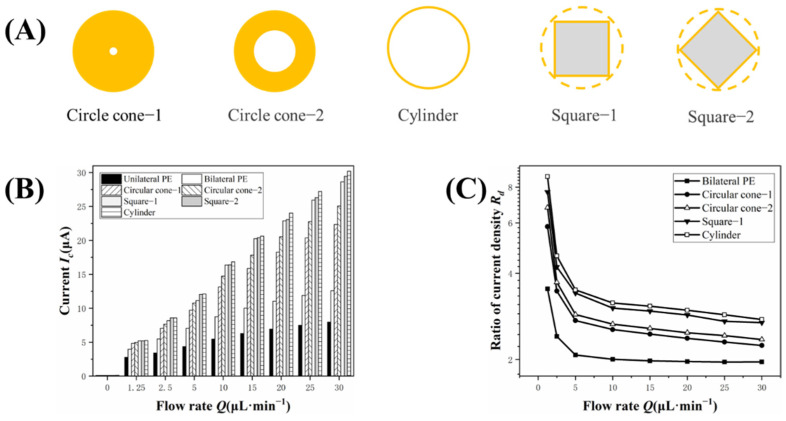
(**A**) Cross-section of the micropillars in different shapes with the same base radius (50 μm). (**B**) Current responses of the PE, the bilateral PE and bilateral μAEs in different shapes under varying flow rates; (**C**) The current density ratios of these μAEs in different shapes under varying flow rates.

The micropillars’ shape affects the surface area of the bµAE and the flow around the electrode. In this section, the influence on the current is studied in terms of shape and relative angles of micropillars to the flow direction. The micropillars in various forms were investigated, including circle cone, cylinder, and square (See Figure 6A). The dimensions of micropillars with different shapes are displayed in Table S2.

As shown in Figure 6B, the electrochemical performance of bµAEs with different shapes of micropillars was investigated. The current responses of bµAEs with all the micropillar shapes were larger than that of planar electrodes. This current increment was particularly pronounced for the bμAE with the cylindrical micropillars. For instance, the bμAE with the Circle cone-Ⅰ and Cylindrical micropillars showed a response current of 2.2 and 2.8 times that of the planar electrode (4.4 μA) at the flow rate of 5 µL·min^−1^, respectively. Additionally, the effects of different relative angles on the current of bµAEs were studied and the results showed that the relative angles have almost no influence on the current. The current of bμAEs with the micropillars of Square-Ⅰ and Square-Ⅱ were almost the same at various flow rates, and the difference in current values was only 4.2% at 30 µL·min^−1^.

Figure 6C shows the effect of different shapes of micropillars on the tail effect. The variation trend of the current density ratio can be expressed as different shapes of the micropillars, leading to the uniform shift in current density ratios, and relative angles have little effect on the tail effect. The difference in tail effects between different shapes is mainly due to the change in total surface area. However, bµAEs with large surface areas cannot yield a large current at low flow rates because the analyte in the microchannel is almost completely consumed. Therefore, it can be concluded that cylindrical micropillars have the advantage of an increased surface area, and disable to obtain a large current at low flow rates due to tail effects.

### 3.5. Experimental Verification

**Figure 7:**
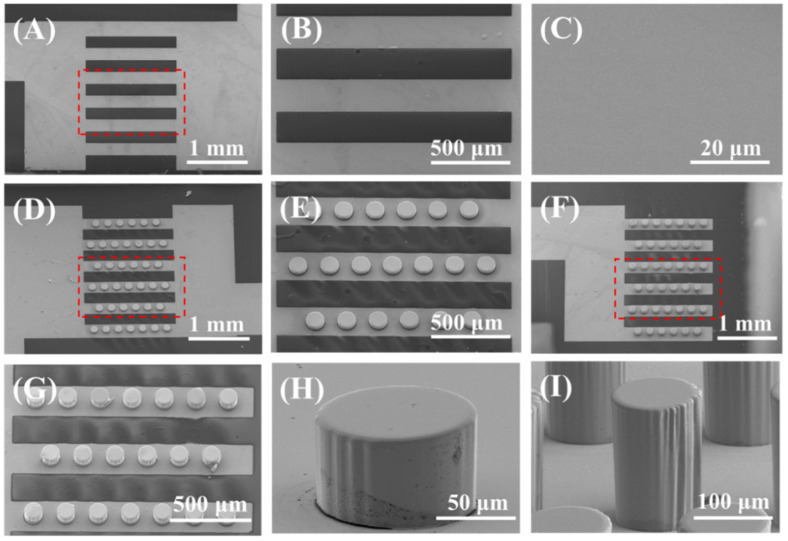
The SEM images of the planar electrode, the developed electrode bottom layer and the electrode top layer. (**A**–**C**) the top view and the enlarge view of the planar electrode. (**D**,**E**) the top view and the enlarge view of the bottom electrode layer with the 50−μm micropillars. (**F**,**G**) the top view and the enlarge view of the top electrode layer with the 150−μm micropillars. (**H**,**I**) the single micropillar with heights of 50 μm and 150 μm.

The SEM images of the bμAEs and the planar electrode are compared in Figure 7. There are no other impurities or contaminations on the surface of electrodes, illustrating that bμAEs were successfully manufactured after the standard micromachining fabrication. We fabricated two kinds of bμAEs with different heights, namely, 50 and 150 μm. The height of micropillars on the cross-sectional images of the bμAE with 50 μm and 150 μm micropillars were measured as 46.3 ± 6.4 μm and 155.8 ± 12.9 μm, respectively. The bottom radius of the bμAE with 50 μm and 150 μm micropillars were measured as 112.5 ± 10.3 μm and 109.1 ± 3.8 μm, respectively, while the center-to-center distance between two pillars within the same row or column was measured around 200 μm. The bμAEs with 150 μm micropillars are used for the following verification experiment.

**Figure 8:**
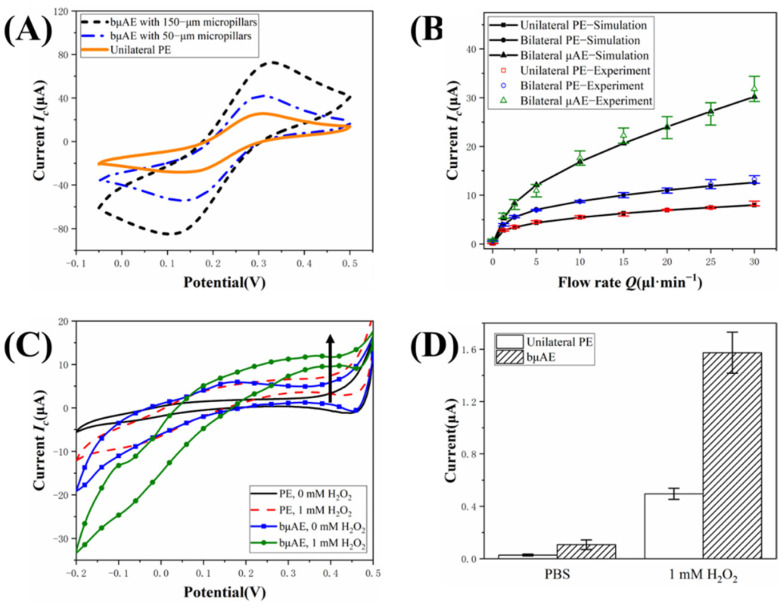
(**A**) CV of the unilateral planar electrode, the bμAE with 50−μm micropillars and 150−μm micropillars in the solution containing 5 mM K_3_[Fe(CN)_6_]/K_4_[Fe(CN)_6_] and 0.1 M KCl.; (**B**) Experimental and simulated current response of the unilateral planar electrode, the bilateral planar electrode and the bμAE with 150− μm micropillar at different flow rates. (**C**) CV of the unilateral planar electrode and the bμAE with 150−μm micropillars at different H_2_O_2_ concentrations. (**D**) Comparison of current response between the planar and the bμAE with 150−μm micropillars toward different H_2_O_2_ concentrations when the flow rate is 1 µL·min^−1^.

The cyclic voltammetry was used in a standard redox system to understand the electrochemical activity of the PE and the bμAEs, as shown in Figure 8A. It can be seen that the CV curves of the bμAE with 50 μm and 150 μm micropillars show a more prominent peak current than the planar electrode, illustrating the large electroactive area of the bµAEs. As indicated by the CV curves, +0.4 V was used as the constant potential (versus the RE of AgCl) for the CA analysis. Figure S9 depicts the current response of the unilateral PE, the bilateral PE, and the bμAE with 150 μm micropillar at various flow rates. The current response between t = 40~60 s reached a plateau, and the current at 50 s was recorded as the final equilibrium current and plotted in Figure 8B. The experimental data of the response current are in good agreement with the simulation results, demonstrating the effectiveness and accuracy of the numerical model. 

Hydrogen peroxide (H_2_O_2_) is used as a model analyte to demonstrate the feasibility and advantage of the developed microchip integrated with bμAEs in the biosensing application. The CV curves of the PE and the bμAE with 150 μm micropillars are displayed in Figure 8C toward different H_2_O_2_ concentrations. Based on the CV result, the potential of +0.4 V was chosen as the working potential in the following CA experiments. The pulse width of the CA measurement was set as 60 s, and the current response at 50 s was recorded as the equilibrium current. The analyte was pumped into the microchip at the flow rate of 2.5 µL·min^−1^. As shown in Figure S10, the equilibrium current of both the PE and the bμAE increases with the increase in H_2_O_2_ concentration. Furthermore, the equilibrium current (1.57 ± 0.15 μA) of the microchip integrated with bμAEs is approximately three times that of the PE (0.5 ± 0.04 μA) toward 1 mM H_2_O_2_ (see Figure 8D), illustrating the high sensitivity of the developed microchip. These results demonstrate that the microchip integrated with bμAEs has great potential in the developing sensitive electrochemical sensors.

## 4. Conclusions

In this paper, we first propose a bio-inspired micropillar array electrode based on the microfluidic chip, deriving from the inspiration from leatherback sea turtles’ mouths. It was successfully demonstrated that deforming a μAE to rearrange the micropillars in bilateral sides of the microchannel is a feasible way to improve the performance of a μEDS further. The numerical method was utilized to study the electrochemical performance of the microchip integrated with bμAEs with different geometric parameters (e.g., spacings, heights, layouts and shapes). It was found that the bμAE yields a larger current than the generally used planar electrode and the unilateral μAE. Micropillars of small spacings do not improve the current of bμAEs while the flow rates are low, because of the almost complete reaction of electrodes and reactants. High micropillars lead to a large current response due to the thin diffusion layer around the micropillar of the bμAE, resulting in a better mass transfer. Regarding different layouts, bμAEs with staggered layouts behave better than bμAEs with aligned layouts at a relatively high flow rate. In addition, the bμAE with cylindrical micropillars shows a larger current than that of bμAEs with other shapes (e.g., circle cone and square) of micropillars when the flow rate is high. The tail effect limits the current of downstream micropillars, and increasing the surface area of bμAE through reducing the spacing and increasing the height of micropillars would exacerbate the tail effect. Finally, we prepared a bμAE by microfabrication and verified the numerical model with CA measurements. The results show that the experimental data agree with the simulation results, indicating the working of the proposed bμAE and the good suitability of the numerical model. 

This work has reported a new bionic structure for μAEs, and further guides the optimization of geometric parameters of bμAEs and provides a feasible method for the fabrication of bμAEs. Due to the large current improvement and the high sensitivity, the microfluidic platform integrated with bμAE shows great potential in biosensing, including but not limited to metabolite detection, protein detection and immunoassay.

## Figures and Tables

**Table 1:** Parameters of the bµAEs for numerical study.

Parameters	Planar	μAE		bμAE			
Projection area (mm^2^)	1.5×2.5						
Radius *r* (μm)	-	50	50				
Height *h* (μm)	-	150/250	50/100/150/200/250				
Spacing ^1^ *d* (μm)	-	200	200	200	150/200/250	200	200
Number of pillars *n*	-	78	78	78	136/78/46	78	78
Surface area *S* (mm^2^)	3.8	7.4/9.9	5.0	6.2	10.2/7.4/5.9	8.7	9.9
Area ratio ^2^	1	1.9/2.6	1.3	1.6	2.7/1.9/1.6	2.3	2.6

^1^ The spacing between two adjacent micropillars. ^2^ The ratio of the surface area between the electrode and the planar electrode.

**Table 2:** Boundary conditions used in the simulations.

Parameters	Unit	Value
Diffusion coefficient *D*	cm^2^/s	6.39 × 10^−6^
Faraday’s constant *F*	C/mol	96,485.33
Standard heterogeneous rate constant *k*_0_	m/s	1 × 10^−4^
Transfer coefficient α	-	0.6
Gas constant *R*	J/(mol·K)	8.314
Absolute temperature *T*	K	298.15
Formal potential *E0′*	V	0.15
Applied potential *E*	V	0.4

## Data Availability

Not applicable.

## Supplementary Materials

The following supporting information can be downloaded at: https://www.mdpi.com/article/10.3390/bios12100878/s1, Figure S1: (A) The computational domain of the microchip with bμAE. (B) The diagram of the meshing method; Figure S2: (A) The figure of the real chip. (B) The figure of the microchip based on the adhesion of double-sided tape when the dye solution was injected into the chip at the flow rate of 100 µL·min^−1^. (C) The schematic diagram of the electrochemical detection system.; Figure S3: The schematic diagram of the cross-section (A) and the concentration distribution in this section at different flow rates: (B) Q = 1.25 µL·min^−1^; (C) Q = 10 µL·min^−1^; (D) Q = 30 µL·min^−1^.; Figure S4: The current responses of the three components (i.e., the micropillar, the planar base, and total surface area) of μAE and bμAE with spacings of 200 µm at various flow rates.; Figure S5: The current responses of the first and the last row of micropillars of the bµAE and µAE at different flow rates. The spacing of the bµAE and µAE is 200 μm.; Figure S6: Concentration distribution in the bilateral μAE with different spacings at the flow rate of 10 µL·min^−1^: (A) d = 150 μm; (B) d = 200 μm; (C) d = 250 μm.; Figure S7: The concentration distribution of the bμAEs with 250-μm micropillars at the symmetrical section when the flow rate varies from 1.25 to 15 μL·min^−1^: (A) the flow rate is 1.25 μL·min^−1^; (B) the flow rate is 2.5 μL·min^−1^; (C) the flow rate is 5 μL·min^−1^; (D) the flow rate is 10 μL·min^−1^; (E) the flow rate is 15 μL·min^−1^; Figure S8: The concentration distribution of the bμAEs, μAEs and the planar electrode on the cross-section (z = 75 μm) at the flow rate of 20 μL·min^−1^: (A) planar electrode; (B) The aligned unilateral μAE with 150 μm micropillars; (C) The aligned bilateral μAE with 150 μm micropillars; (D) The staggered bilateral μAE with 150 μm micropillars. (E) Comparison of current of the bμAE with 250 μm micropillars in staggered layout and aligned layout.; Figure S9: Experimental CA of (A) the unilateral planar electrode, (B) the bilateral planar electrode and (C) bilateral μAE150 at different flow rates in the 5 mM K_3_[Fe(CN)_6_]/K_4_[Fe(CN)_6_] solutions with 0.1 M KCl vs. Ag/AgCl; Figure S10: CA curves of (A) the unilateral planar electrode and (B) bμAE with 150 μm micropillars at different H_2_O_2_ concentrations.; Table S1: Parameters of the working electrode with different layouts; Table S2: Parameters of the working electrode with different shapes.
