# Supplementary material for: Numerical Study on a Bio-Inspired Micropillar Array Electrode in a Microfluidic Device
Source: Biosensors (Basel). 2022 Oct 16;12(10):878. doi: 10.3390/bios12100878 (PMC9599680; doi:10.3390/bios12100878)
Supplement: Supplementary file 1 [file biosensors-12-00878-s001.zip › biosensors-1948365-supplementary.pdf]

Numerical Study on a Bio-inspired Micropillar Array Electrode in a Microfluidic Device

Chaozhan Chen[1], Bin Ran[1], Bo Liu[1], Xiaoxuan Liu[1], Jing Jin[2,*] and Yonggang Zhu[2,*]

[1] School of Science, Harbin Institute of Technology, Shenzhen, Shenzhen, 518055, China

[2] School of Mechanical Engineering and Automation, Harbin Institute of Technology, Shenzhen, Shenzhen, 518055, China

[*] Corresponding author.

E-mail address: jinjing2020@hit.edu.cn; zhuyonggang@hit.edu.cn;

(A)

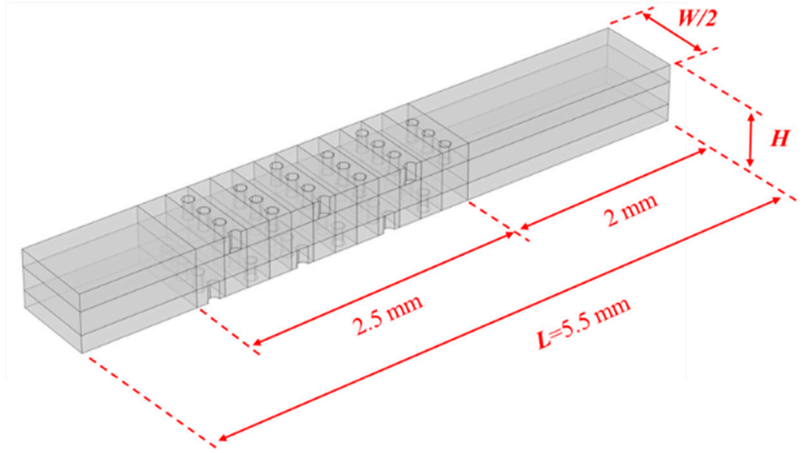

(B)

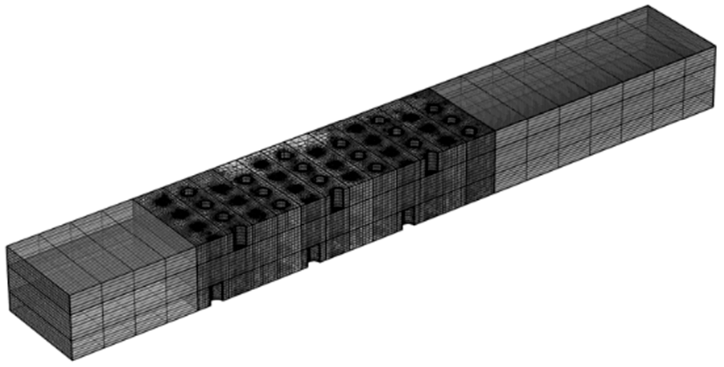

Figure S1. (A) The computational domain of the microchip with bμAE. (B) The diagram of the meshing method.

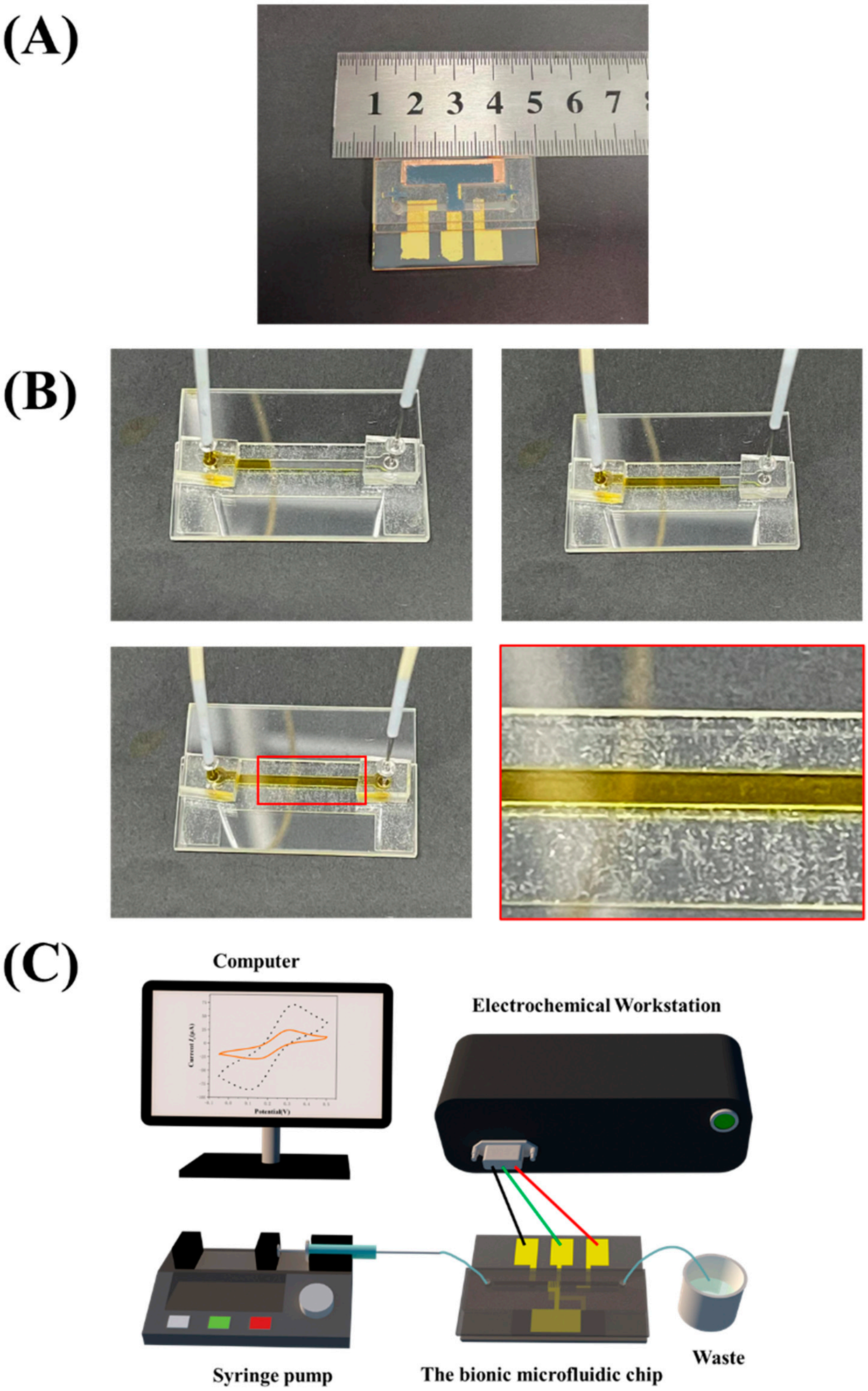

Figure S2. (A)The figure of the real chip. (B) The figure of the microchip based on the adhesion of double-sided tape when the dye solution was injected into the chip at the flow rate of 100 μl.min^{-1}. (C)The schematic diagram of the electrochemical detection system.

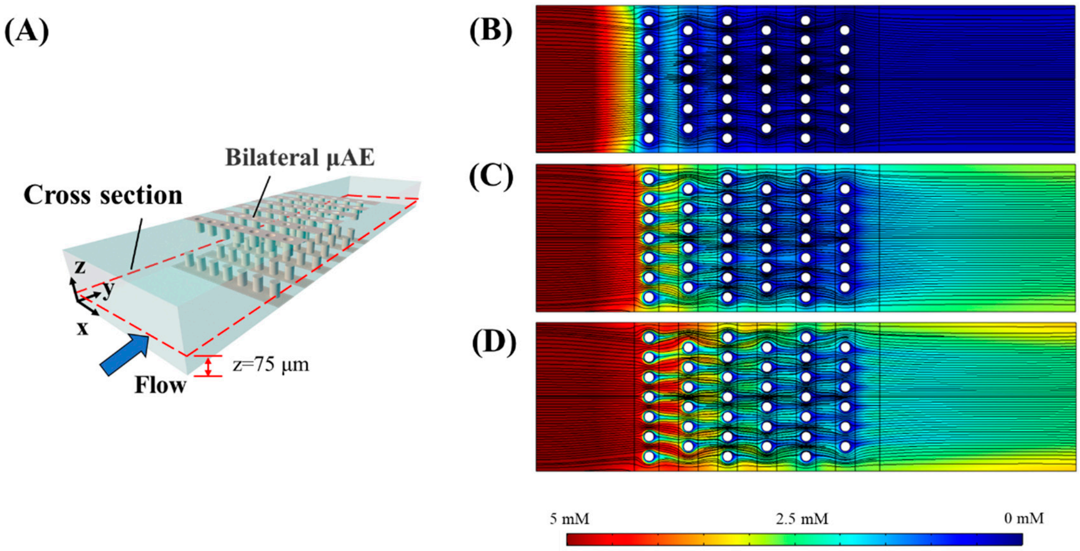

Figure S3. The schematic diagram of the cross section (A) and the concentration distribution in this section at different flow rates: (B) Q=1.25 μL·min^{-1};(C) Q=10 μL·min^{-1}; (D) Q=30 μL·min^{-1}.

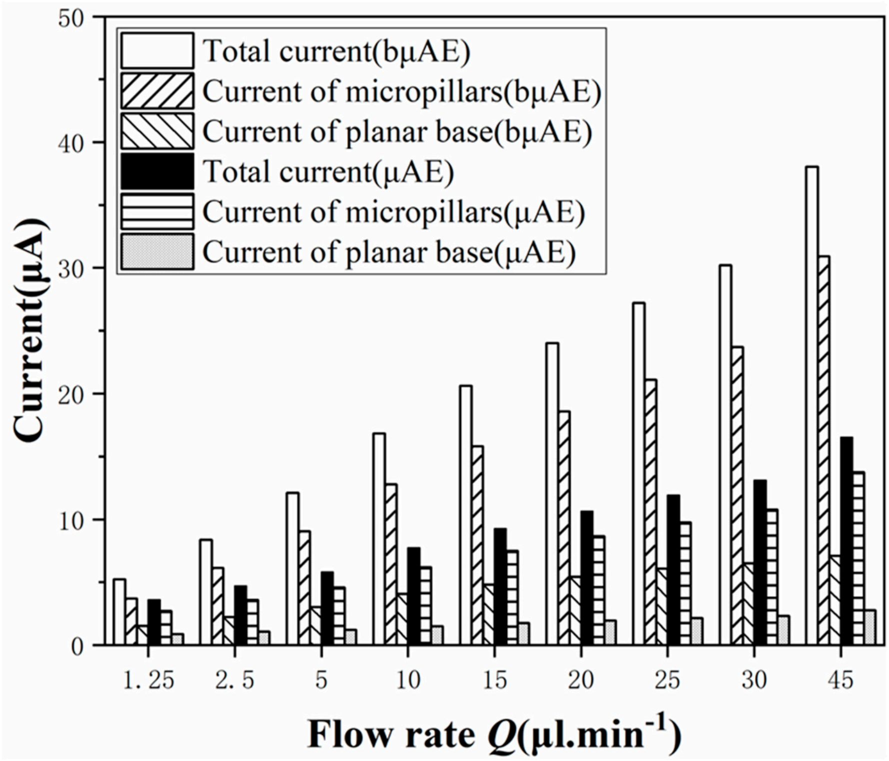

Figure S4. The current responses of the three components (i.e., the micropillar, the planar base, and total surface area) of μAE and bμAE with spacings 200 μm at various flow rates.

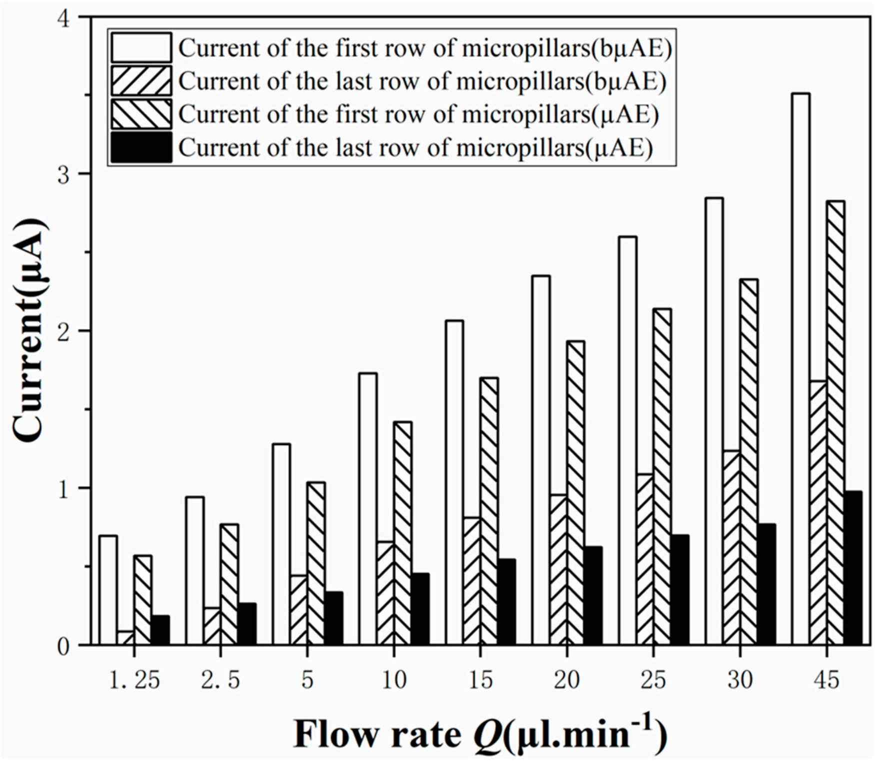

Figure S5. The current responses of the first and the last row of micropillars of the bμAE and μAE at different flow rates. The spacing of the bμAE and μAE is 200 μm.

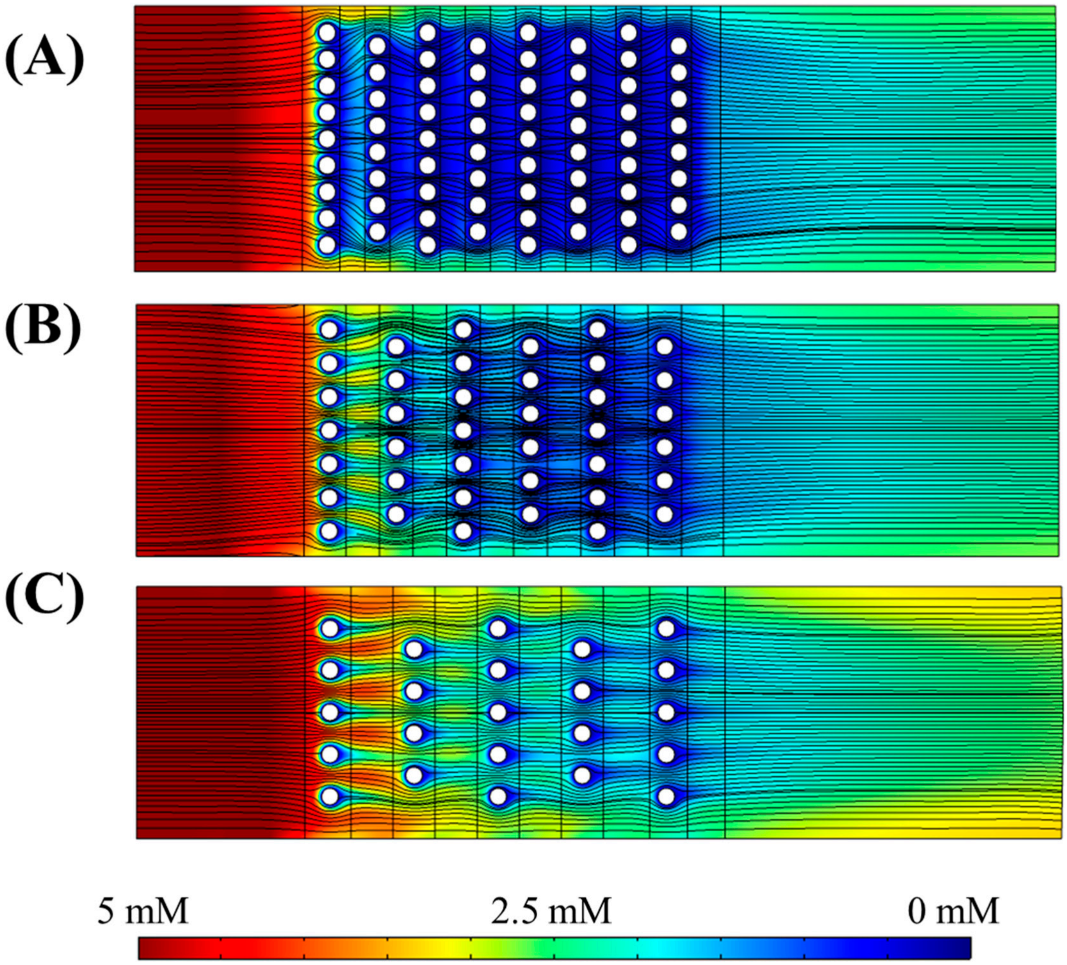

Figure S6. Concentration distribution in the bilateral μAE with different spacings at the flow rate of 10 μL·min^{-1}: (A) d=150 μm; (B) d=200 μm; (C) d=250 μm.

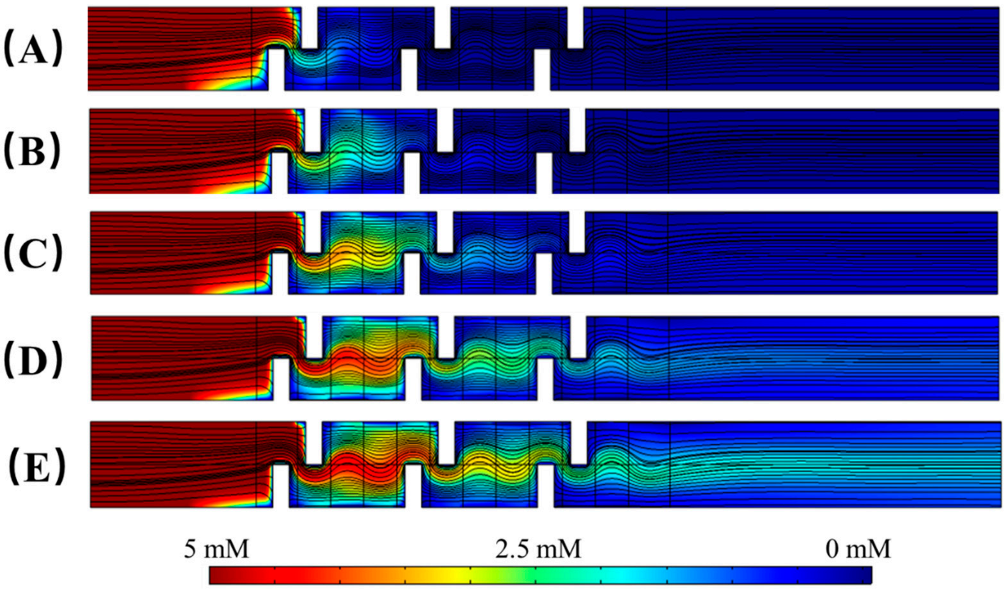

Figure S7. The concentration distribution of the bμAEs with 250-μm micropillars at the symmetrical section when the flow rate is varies from 1.25 to 15 μL·min^{-1}: (A) the flow rate is 1.25 μL·min^{-1}; (B) the flow rate is 2.5 μL·min^{-1}; (C) the flow rate is 5 μL·min^{-1}; (D) the flow rate is 10 μL·min^{-1}; (E) the flow rate is 15 μL·min^{-1}

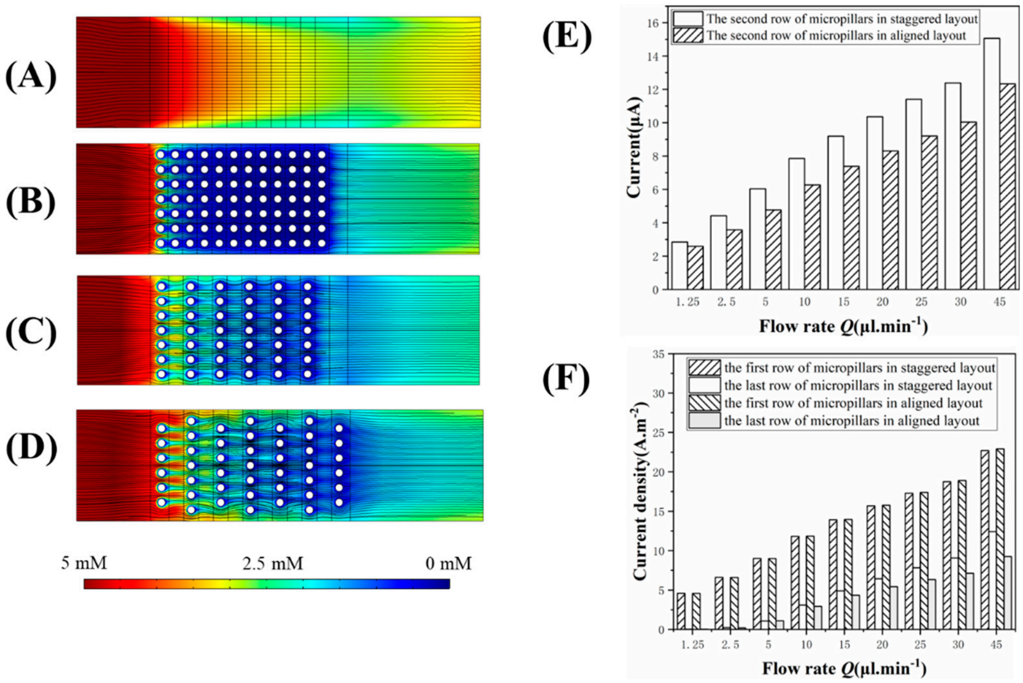

Figure S8. The concentration distribution of the bμAEs, μAEs and the planar electrode on the cross section (z=75 μm) at the flow rate of 20 μL·min^{-1}: (A) planar electrode; (B) The aligned unilateral μAE with 150-μm micropillars; (C) The aligned bilateral μAE with 150-μm micropillars; (D) The staggered bilateral μAE with 150-μm micropillars. (E) Comparison of current of the bμAE with 250-μm micropillars in staggered layout and aligned layout. (F) The current of bμAEs with 250-μm micropillars in staggered layout and aligned layout

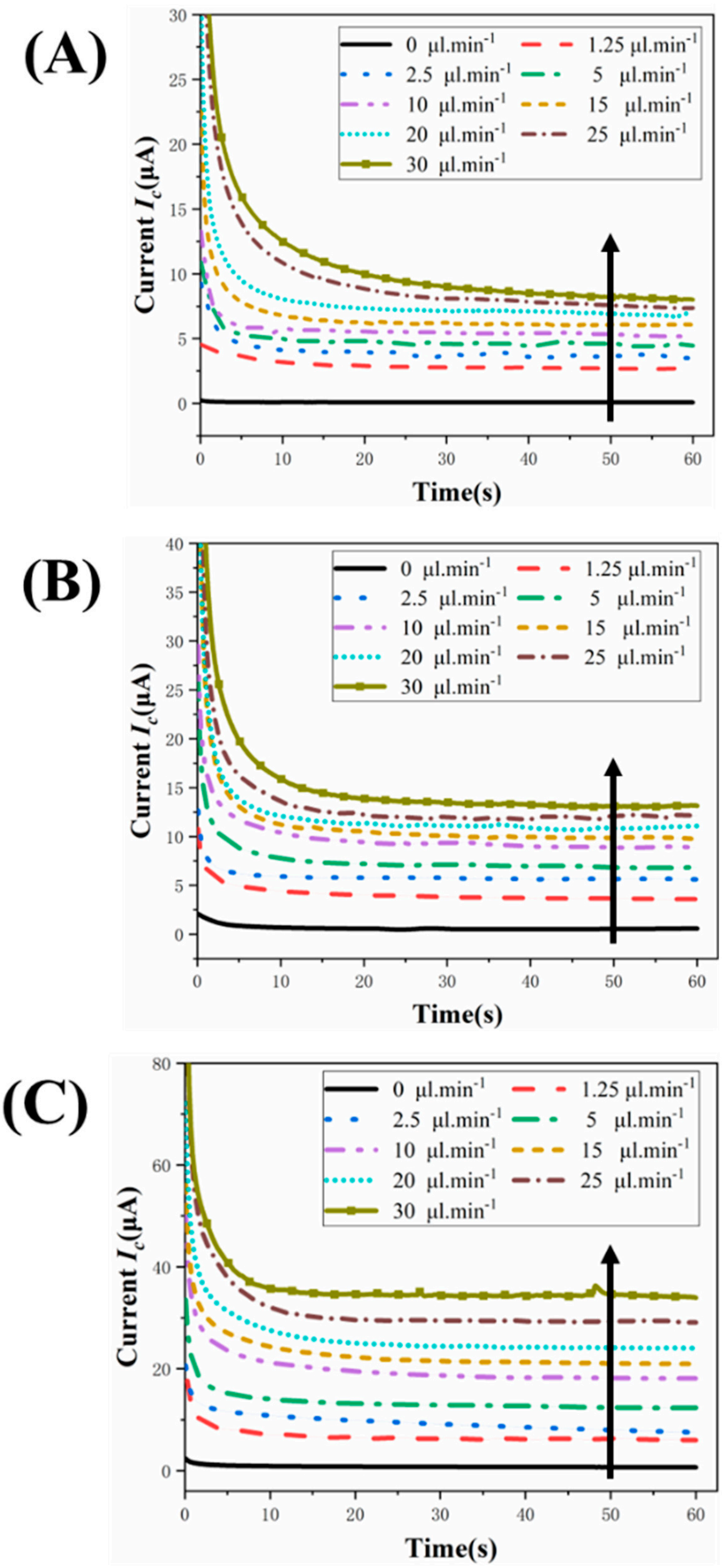

Figure S9. Experimental CA of (A) the unilateral planar electrode, (B) the bilateral planar electrode and (C) bilateral μAE150 at different flow rates in the 5 mM $K_3[Fe(CN)_6]/K_4[Fe(CN)_6]$ solutions with 0.1 M KCl vs Ag/AgCl.

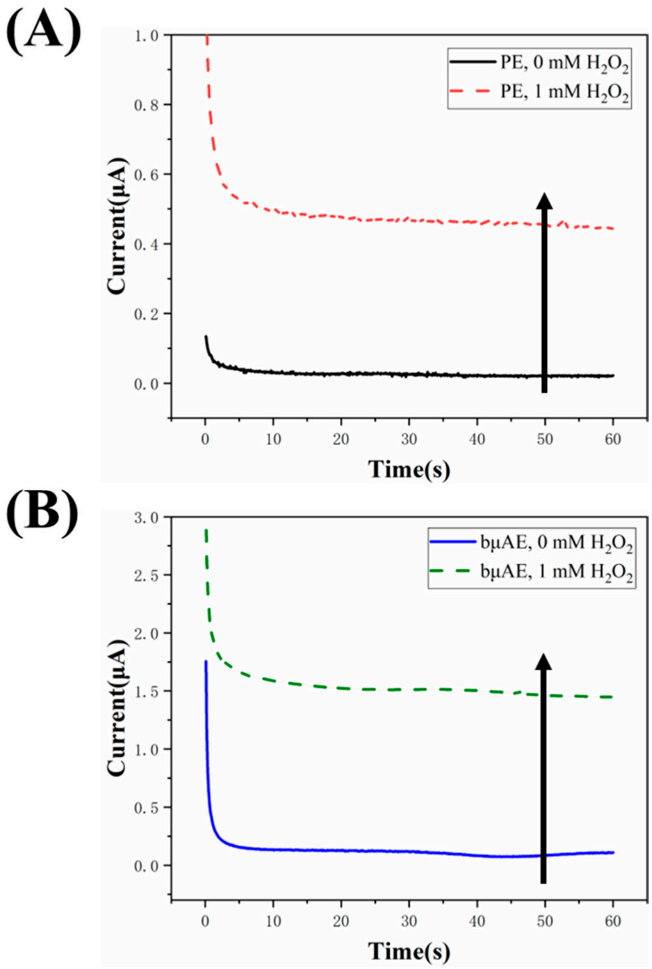

Figure S10. CA curves of (A) the unilateral planar electrode and (B) bμAE with 150-μm micropillars at different H_2O_2 concentrations.

Table S1. Parameters of the working electrode with different layout.

Parameters	Planar	Aligned unilateral μAE	Aligned bμAE-1	Staggered bμAE -1	Aligned bμAE-2	Staggered bμAE-2
Projection area $l \times w$ (mm^2)	1.5×2.7					
Height h (μm)	-	150	150	150	250	250
Spacing d (μm)[1]	-	200				
Number of pillars n	-	84	84	84	84	84
Pillar radius r_p (μm)	-	50	50	50	50	50
Surface area S (mm^2)	4.05	7.72	7.72	7.72	10.17	10.17
Layout type	-	Aligned	Aligned	Staggered	Aligned	Staggered
Area ratio[2] S_g	1	1.91	1.91	1.91	2.51	2.51

[1]Spacing between the centers of two adjacent micropillars.
[2]The ratio of the surface area between the μAE and the planar electrode.

Table S2. Parameters of the working electrode with different shapes.

Parameters	Planar	Circle cone-1	Circle cone-2	Square-1	Square-2	Cylinder
Projection area $l \times w$ (mm^2)	1.5×2.5					
Height h (μm)	-	150				
Spacing d (μm)[1]	-	200				
Number of pillars n	-	78				
Top radius r_t (μm)	-	12.5	25	-	-	50
Base radius (μm)	-	50	50	-	-	50
Side length (μm)	-	-	-	70.71	70.71	-
Surface area S (mm^2)	3.75	4.97	6.06	7.03	7.03	7.42
Layout type	-	Staggered	Staggered	Staggered	Staggered	Staggered
Area ratio[2] S_g	1	1.33	1.62	1.87	1.87	1.98

[1]Spacing between the centers of two adjacent micropillars.

[2]The ratio of the surface area between the μAE and the planar electrode.
